# Medical ethics: knowledge, attitude and practice among doctors in three teaching hospitals in Sri Lanka

**DOI:** 10.1186/s12910-020-00511-4

**Published:** 2020-08-05

**Authors:** A. W. I. P. Ranasinghe, Buddhika Fernando, Athula Sumathipala, Wasantha Gunathunga

**Affiliations:** 1 Postgraduate Institute of Medicine, University of Colombo, Colombo, Sri Lanka; 2 Research Institute for Primary Care and Health Sciences, School of Medicine, Keele University, Keele, UK; 3 Faculty of Medicine, University of Colombo, Colombo, Sri Lanka

**Keywords:** Doctors, Medical ethics, Knowledge, Attitudes, Practice, Sri Lanka

## Abstract

**Background:**

Medical ethics deals with the ethical obligations of doctors to their patients, colleagues and society. The annual reports of Sri Lanka Medical Council indicate that the number of complaints against doctors has increased over the years. We aimed to assess the level of knowledge, attitude and practice regarding medical ethics among doctors in three teaching hospitals in Sri Lanka.

**Methods:**

A hospital-based cross-sectional study was conducted among doctors (*n* = 313) using a pre-tested self-administered, anonymous questionnaire. Chi Squared test, and ANOVA test were used to identify the significance of association between level of knowledge and selected factors.

**Results:**

Most doctors (81.2%) had a poor level of knowledge on medical ethics, with postgraduate trainees showing significantly (*p* = 0.023, Chi square) higher level of knowledge. The average knowledge on medical ethics among doctors was significantly different between the three hospitals (*p* = 0.008, ANOVA).

Over 95% had a favourable attitude towards gaining knowledge and advocated the need for training. The majority (69.3%) indicated awareness of unethical practices. 24.6% of respondents stated that they get a chaperone ‘sometimes’ during patient examination while 3.5% never do. The majority (54%) responded that they never accept gifts from pharmaceutical companies in recognition of their prescribing pattern. 12–41% of doctors participated in the study acknowledged that they ‘sometime’ engaged in unethical practices related to prescribing drugs, accepting gifts from pharmaceutical companies and when obtaining leave.

**Conclusion:**

Most doctors had a poor level of knowledge of medical ethics. Postgraduate trainees had a higher level of knowledge than other doctors. The majority showed a favourable attitude towards gaining knowledge and the need of training. Regular in-service training on medical ethics for doctors would help to improve their knowledge on medical ethics, as well as attitudes and ethical conduct.

## Background

Sri Lanka is a middle-income country that provides free health services to all citizens despite many challenges, and as a result, has one of the better health indicator profiles amongst Asian countries [1, 2]. Healthcare personnel of all categories, including doctors, have contributed immensely to achieve this status. Whilst the country has gained acclaim for these better health indicators, there has been a recent increase in the public outcry against health care professionals, especially doctors [3], with an increase in the number of complaints lodged against doctors at the Sri Lanka Medical Council (SLMC), the statutory body in Sri Lanka that licenses doctors [4]. Almost all complaints are related to alleged breaches of one or more of the four basic principles of medical ethics: respect for autonomy, non-maleficence, beneficence and justice. A copy of the guidelines on ethical conduct for medical and dental practitioners [5] compiled by the Ethics Committee of the SLMC is given to doctors at the time of registration. The SLMC expects all members of the medical profession to adhere to these guidelines.

Ethical principles help identify what is considered right or wrong at a given time, in a given culture, in line with the perceived moral consequences of the action [6]. Medical ethics deals with the ethical obligations of doctors to their patients, colleagues and society [7]. Ethics often prescribe a higher standard of behaviour than does the law [8]; something that is legal is not necessarily moral or ethical.

Criticism from the media [9] and litigation, though not necessarily supported by evidence of ethical misconduct, are a major challenge for doctors. On the other hand, many incidents of ethical misconduct by doctors do not appear in the media and courts, due perhaps to the tradition of respect towards doctors or the power imbalance between doctors and patients. However, even a few incidents of professional misconduct can significantly affect the entire health system in the country, occasionally even leading to violence and trade union action.

Adequate knowledge, favourable attitude and comprehensive training on medical ethics help prepare medical professionals to anticipate, cope with, discuss and resolve ethical dilemmas and challenges encountered in day-to-day practice. Medical ethics teaching as well as practice is necessary to achieve the competencies necessary to deliver services in an ethical manner [10]. The ethics component of the undergraduate medical curricula has therefore been revised and improved over the past few decades with changes based on the needs of the community [11].

A previous literature search was updated to find out the current evidence on the knowledge, attitude and practice of medical ethics among doctors in Sri Lanka. A Pub Med search with the keywords “medical ethics”, “Sri Lanka” and “doctors” (02 July 2019) generated two hits but neither was relevant to this specific subject matter. No articles of the same theme originating from Sri Lanka were found on a Google Scholar search or a search among medically related journals hosted on the Sri Lanka Journals Online platform on the same date. A few studies to assess knowledge, attitude and practice on medical ethics in neighbouring South Asian countries indicate the need to improve doctors’ knowledge of ethics [12, 13]. A study conducted in India found that although most doctors (98.7%) had heard of the code of ethics, only 59.7% had read it, and only one-third of respondents (95/315; 30.1%) had adequate knowledge on the code of ethics [12]. In Bangladesh it was found that the standards of public health are gradually deteriorating following the commercialization of services by private doctors; advertising, recruitment of agents to get more patients, unnecessary surgeries and overcharging have become major ethical problems [14].

Lack of evidence is one of the main obstacles for rational interventions. In the background of increasing complaints against doctors as evidenced by the annual reports of the SLMC [4], this study was designed to assess the knowledge, attitude and practice of medical ethics among doctors and to describe factors associated with the level of knowledge on medical ethics in three tertiary care teaching hospitals in the Kandy district of Sri Lanka in 2009.

## Methodology

### Study setting

A hospital based descriptive cross-sectional study was conducted in three tertiary care teaching hospitals, General Hospital Kandy (GHK), Teaching Hospital Peradeniya (THP) and Sirimavo Bandaranaike Specialised Children Hospital Peradeniya (SBSCH), in the Kandy district of Sri Lanka in 2009. Over 900 doctors of all grades work in these three tertiary care level (teaching) hospitals.

### Study participants

Study participants were medical doctors who worked in these three hospitals with full registration with the SLMC. Respondents were selected based on convenience sampling due to feasibility constraints. Intern House Officers were excluded as they were trainees and did not have full registration of the SLMC. The doctors affiliated to the university were excluded as they represented a different category of professionals who were under the administration of Department of Higher Education. Medical Officers who had been released for training or were on leave for a long period (e.g. maternity leave, leave for overseas training), were excluded.

### Sample size

The sample size was calculated using the formula developed by Lwanga and Lameshow [15] to estimate a population proportion. At a precision of 0.06, the proportion of good level of knowledge at 50%, and at a confidence level of 95% with an expected non- response rate of 15%, the calculated sample size was 314.

### Study instrument

A structured self-administered questionnaire was developed with judgmental validity (face, content and consensual validity) and pre-tested.

The questionnaire consisted of 25 questions of true/false nature on different aspects of knowledge of prescribed guidelines on medical ethics for practising doctors in Sri Lanka. The Guidelines on ethical conduct for medical and dental practitioners registered with the Sri Lanka Medical Council (SLMC) were studied in great detail and finally used as the basis for developing questions which appears in the questionnaire (supplementary material 1). Priority was given to ethical issues commonly encountered by many doctors in Sri Lanka. Issues regarding making decisions near the end of life, euthanasia, organ donation and transplant were not included as these issues are not yet frequently encountered by majority of doctors.

Attitude towards medical ethics was assessed using 15 attitudinal statements with responses given on a five-point Likert scale (Table 3). The extent of ethical medical practice was assessed using statements and questions with responses given according to the frequency of practices on a four-point rating scale (Table 4).

### Ethics approval and consent to participation

Participation in the study was purely on a voluntary basis with informed verbal consent. During pre-testing of the study instrument, it was evident that many doctors preferred to avoid giving written consent even after they were informed that the data would be anonymised, since the questionnaire contained questions about their conduct, which made this study highly sensitive. We obtained ethics review and approval from two local ethics review committees (Ethical Review Committee, Faculty of Medicine, University of Peradeniya, Sri Lanka and the Ethical Committee, Postgraduate Medical Centre, General Hospital (Teaching), Kandy, Sri Lanka) in addition to the initial ethics approval granted by the Ethical Review Committee, Faculty of Medicine, University of Kelaniya, Sri Lanka. Approval was sought from all relevant institutions due to the sensitive nature of the questions asked. All three ERCs approved the study methodology including informed verbal consent process for the study.

Furthermore, permission to conduct the research was obtained from relevant hospital directors prior to data collection.

### Data collection

The questionnaire was enclosed in a sealed, unmarked envelope and administered to all eligible and accessible participants. Data was collected out of working hours, such as tea breaks and lunch breaks, during working days, ensuring that routine work was not interrupted. To ensure confidentiality, the questionnaires were completed anonymously and collected into sealed boxes which had been kept in places where it was convenient to them. Participants were requested to answer the questionnaire and to return it on the same day. Those who could not find time due to many practical reasons to fill the questionnaire same day, were given an additional day to respond. Participants were given a reminder by the principle investigator and those who did not respond even after the third reminder was considered non-respondents. The principle investigator distributed the questionnaire and the same set of information was given to all participants approached for the study.

### Data analysis

Each question testing knowledge with a correct answer was given four marks, giving a total of 100 marks. In the analysis, a knowledge score of 60 or above was considered as a good level of knowledge since the questions asked were related to routine clinical practice and the availability of guidelines. The 15 statements on attitude were assessed by a five-point Likert scale. The12 practice related statements were assessed using a rating scale. Percentages were calculated for each of the options. The Chi Squared test and ANOVA test were used to identify the significance of association between level of knowledge and selected factors. Distribution of the knowledge score was plotted to check if the data was normally distributed before the ANOVA test was applied.

## Results

Of a total eligible participant population of 946 at the three sites, 502 were contacted and 313 responded - a response rate of 62%.

160 (51.1%) of the respondents were male and 50.2% were aged 35–44 years. The majority was Sinhala (89.5%) and Buddhist (87.5%). There were 66.8% Grade II doctors and 5.1% specialist doctors. Two-thirds of the respondents (68.7%) had worked less than 9 years. Study participants were predominantly from medical (20.1%), surgical (17.6%), paediatric (15%) and outpatient (12.8%) departments. 19.5% of the study participants were postgraduate trainees. Table 1 shows the socio-demographic characteristics of the study population.

**Table 1:** Socio-demographic characteristics of the study population

Characteristic	Frequency (*n* = 313) N, %
Sex	
Male	160 (51.1%)
Female	153 (48.9%)
Age	
24–34	112 (35.8%)
35–44	157 (50.2%)
45–54	32 (10.2%)
≥55	12 (3.8%)
Ethnicity	
Sinhala	280 (89.5%)
Tamil	12 (3.8%)
Moor	19 (6.1%)
Other	2 (0.6%)
Religion	
Buddhist	274 (87.5%)
Hindu	13 (4.2%)
Roman Catholic/Christian/Protestant	4 (1.3%)
Islam	19 (6.1%)
Other	3 (1.0%)
Civil Status	
Married	272 (86.9%)
Single	39 (12.5%)
Divorced	2 (0.6%)
Professional Status	
Specialist medical officers	16 (5.1%)
Medical Officers (Grade I)	40 (12.8%)
Medical Officers (Grade II)	209 (66.8%)
Medical Officers (Preliminary Grade)	48 (15.3%)
Workstation	
General Hospital Kandy	187 (59.7%)
Teaching Hospital Peradeniya	66 (21.1%)
Sirimavo Bandaranaike Specialized Children’s Hospital	60 (19.2%)
Years in service	
0–4	90 (28.8%)
5–9	125 (39.9%)
10–14	47 (15.0%)
15–20	27 (8.6%)
> 20	24 (7.7%)
Specialty	
Administration	5 (1.6%)
Medical Units	63 (20.1%)
Surgical Units	55 (17.6%)
Paediartics	47 (15.0%)
Obstetrics & Gynaecology	8 (2.6%)
OPD	40 (12.8%)
Anaesthesia	31 (9.9%)
Psychiatry	13 (4.2%)
Other Units	51 (16.3%)
Current postgraduate straining status	
Postgraduate trainees	61 (19.5%)
Non-trainees	252 (80.5%)

The minimum individual knowledge score was 12 while the maximum was 88. The mean score on knowledge was 49.83 (SD13.59). A clear majority (81.2%) had a ‘poor’ knowledge (a score less than 60 out of 100) on medical ethics. Table 2 shows the distribution of overall level of knowledge on medical ethics.

**Table 2:** Distribution of the study population by overall level of knowledge

Overall level of knowledge	Frequency	Percentage
‘Poor’	254	81.2
‘Good’	59	18.8
Total	313	100.0

Responses to 15 statements towards medical ethics were shown in the Table 3. Most doctors (69.3%) did not agree with the statement that ‘At present the extent of ethical medical practice among doctors is satisfactory’. 91% had the opinion that ‘extent of teaching on medical ethics in undergraduate curriculum is not adequate’. Nearly one-fourth (22.4%) did not agree with ‘under no circumstances does a doctor have the right to shout at a patient ’ while 95.3% expressed the necessity of in-service training in medical ethics.

**Table 3:** Attitude towards medical ethics

Attitude (n-313)	Strongly agree or agree	No opinion	Disagree or strongly disagree
**Ethical principles**			
a. Doctors should make certain that their actions do not intentionally harm another even to a small degree.	284 (90.7%)	23 (7.3%)	6 (1.9%)
b. Doctors should never harm another person physically or psychologically	278 (88.8%)	18 (5.8%)	17 (5.4%)
c. Doctors should not perform an action which might in anyway threaten the dignity of another individual	278 (88.8%)	19 (6.1%)	16 (5.1%)
d. Doctors should treat patients as they would wish others to treat them if they were the patients	253 (80.8%)	41 (13.1%)	19 (6.1%)
e. Under no circumstance, a doctor has right to shout at a patient.	218 (69.6%)	25 (8%)	70 (22.4%)
f. An emotional or sexual relationship with a patient (or with a member of the patient’s family), even with consent, is unethical	244 (78%)	42 (13.4%)	27 (8.6%)
**Ethics in practice**			
a. At present the extent of ethical medical practice among doctors is satisfactory	61 (19.5%)	35 (11.2%)	217 (69.3%)
b. The quality of the service of doctor in the government hospital is negatively affected by his/her private practice	118 (37.7%)	57 (18.2%)	138 (44.1%)
c. Strikes done by doctors are indirectly beneficial to patients	105 (33.5%)	73 (23.3%)	135 (43.1%)
**Training**			
a. In my opinion, the extent of teaching on medical ethics in undergraduate curriculum is not adequate	285 (91.1%)	10 (3.2%)	18 (5.7%)
b. In-service training on medical ethics is a necessity for doctors	298 (95.2%)	7 (2.2%)	8 (2.5%)
**Controversies**			
a. Juniors tend to follow their consultants’ attitudes towards patient care	248 (79.2%)	14 (4.5%)	51 (16.3%)
b. Favouritism for students in medical exams is rare	81 (25.9%)	41 (13.1%)	191 (61%)
c. Abortion should be legalized in Sri Lanka	148 (47.3%)	61 (19.5%)	104 (33.2%)

Responses to 12 statements regarding the extent of ethical medical practice were shown in the Table 4. The practice of writing the generic name of the drug with its brand name was described as ‘sometimes’ by 40.6 and 6.7% as ‘never’. 30.7% of doctors stated that they engage in CME activities ‘sometimes’ whereas 4.5% stated as ‘never’. The proportions of doctors who followed the exact rules and regulations ‘always’ was only 38%. Acceptance of gifts from pharmaceutical companies, given in recognition of one’s prescribing pattern, was acknowledged by 8% of doctors as ‘often’ and 37.7% as ‘sometimes’.

**Table 4:** The extent of ethical medical practice

Practices (*n* = 313)	Always	Often	Sometimes	Never
	No (%)	No (%)	No (%)	No (%)
1. I treat every patient considerately	187 (59.7%)	121 (38.7%)	3 (1.0%)	2 (0.6%)
2. When examining a patient, I get a chaperone	106 (33.9%)	119 (38.0%)	77 (24.6%)	11 (3.5%)
3. I spend enough time to explain the nature, purpose and possible consequences of treatment or procedure when obtaining informed consent from patients	117 (37.4%)	142 (45.4%)	54 (17.2%)	0
4. When prescribing drugs in their brand names, I write the generic name also	56 (17.9%)	109 (34.8%)	127 (40.6%)	21 (6.7%)
5. As a medical officer, I dress appropriately	157 (50.2%)	137 (43.8%)	16 (5.1%)	3 (1.0%)
6. I engage in Continuous Medical Education (CME) activities	70 (22.4%)	133 (42.5%)	96 (30.7%)	14 (4.5%)
7. When taking leave, I follow the exact rules and regulations	119 (38.0%)	152 (48.6%)	40 (12.8%)	2 (0.6%)
8. I engage in private medical practice during normal working hours	0	2 (0.6%)	4 (1.3%)	307 (98.1%)
9. Do you influence patients directly or indirectly to accept private treatment	1 (0.3%)	1 (0.3%)	101 (32.3%)	210 (67.1%)
10. When you come across an instance of professional misconduct, do you bring it to the notice of the medical council?	9 (2.9%)	26 (8.3%)	121 (38.7%)	157 (50.2%)
11. Do you accept gifts from pharmaceutical companies, given in recognition of your prescribing pattern?	1 (0.3%)	25 (8.0%)	118 (37.7%)	169 (54.0%)
12. When managing patients, I consider patient’s religious and cultural views	58 (18.5%)	106 (33.9%)	102 (32.6%)	47 (15.0%)

### Factors associated with level of knowledge on medical ethics

The association between overall level of knowledge on medical ethics and selected factors (sex, age, religion, marital status, duration of service, postgraduate training status, specialty, the institution and the designation) was assessed and the results are shown in Table 5. Postgraduate training was significantly associated with a good level of knowledge (*p* = 0.02). In addition, there was a significant difference of mean knowledge scores between the three teaching hospitals (*p* = 0.008, ANOVA).

**Table 5:** Factors associated with level of knowledge on medical ethics

	Level of knowledge				***P value*** (χ^**2**^)*
Factors (*n* = 313)	Poor		Good		
	No	%	No	%	
**Age** (Years)					
24–44	220	81.8	49	18.2	0.48
45–60	34	77.3	10	22.7	
**Work experience** (years)					
0–14	213	81.3	49	18.7	0.88
≥15	41	80.4	10	19.6	
**Marital status**					
Married	219	80.5	53	19.5	0.46
Unmarried	35	85.4	6	14.6	
**Postgraduate training status**					
Postgraduate trainees	24	39.3	37	60.7	**0.02**
Not postgraduate trainees	140	55.6	112	44.4	
**Religion**					
Buddhist	146	53.1	129	46.9	0.51
Non-Buddhist	18	47.4	20	52.6	
**Private practice**					
Engaged in private practice	43	50.0	43	50.0	0.60
Not engaged in private practice	121	53.3	106	46.7	

* Chi square test

## Discussion

The response rate of the present study was 62% (313/502), whereas a relatively similar study conducted in a district of western Sri Lanka 5 years after the study discussed here reported a response rate of 79% [16]. Another study carried out in a teaching hospital in Manipur, India [12] had a response rate of 78.1% (315/403). The relatively low response rate of the present study may be due to the sensitive nature of the questions asked, especially regarding the medical practice; it may also be due to the lack of time to complete the questionnaire which took 20 min on average. In any case, doctors have been well-recognized in surveys as a professional group with low response rates [17].

### The level of knowledge on medical ethics

This study found that 81.2% of doctors had poor knowledge of medical ethics. A study conducted 5 years after the study under discussion (in 2014), to assess the knowledge and perception of selected aspects of medical ethics and medico-legal duties among medical officers in a district of Sri Lanka (Kaluthara District) reported that 52.2% had a poor level of knowledge, 43.9% had a fair level whereas only 3.9% had a good level of knowledge [16]. In both these studies, the majority of doctors had a poor level of knowledge on medical ethics even though they were all provided with a copy of the ethical guidelines upon registration at the SLMC. This indicates that the mere provision of guidelines does not serve the purpose. Many doctors have probably neither read it nor understood its contents. Poor knowledge of medical ethics could have contributed to the increase of complaints against doctors for alleged unethical medical practices. It is our considered opinion that an adequate mechanism for 360^0^ appraisals prior to the revalidation of the license to practice should be introduced in Sri Lanka to induce doctors to self-update their knowledge on ethics (as much as their clinical knowledge). However, having a higher level of knowledge on ethical guidelines per se is not a guarantee of ethical conduct.

Studies conducted in India and Pakistan reported a similar situation. For example, a study conducted in Manipur found a poor level of knowledge on ethics in 70% of the participants, whereas a study undertaken by Shirazi et al. in Pakistan reported that the knowledge of medical ethics and its application in surgical wards was extremely poor [12, 13]. All these studies used self-administered questionnaires and the questions were based on the codes of ethics developed by the medical councils of the respective countries. These findings indicate that improving medical education on medical ethics is a timely necessity not only for Sri Lanka but for some other south Asian countries as well.

The teaching of medical ethics in Sri Lanka was considered “thoroughly inadequate” [18] in the 1990’s. Nearly two decades later, in this study, 91% of participants agreed that the undergraduate curriculum on medical ethics is inadequate. Over 70% of the participants had graduated five or more years before this study was conducted. Their perceptions may be different from that of newly graduated doctors who may have benefited from the global movement to include more teaching on medical ethics in the medical curriculum in the recent past [19]. The results of this study can be used as a baseline to assess whether subsequent changes in undergraduate and postgraduate curricula have made a difference. Based on our recent review of literature, and to the best of our knowledge, there has been no similar study conducted in Sri Lanka for the last 10 years.

### Attitude towards medical ethics

Most doctors (69.3%) believed that the extent of ethical medical practice at present is not satisfactory, necessitating further exploration of the reasons driving this perception of the professional conduct of doctors. A study conducted in the UK among newly qualified doctors revealed that they were aware of “ethical erosion in themselves and their colleagues” [20]. This indicates the possibility of many doctors being aware of unethical practice. Moreover, 95.3% doctors in the present study identified in-service training on medical ethics as a necessity, indicating a positive response to interventions that can enhance knowledge and attitudes for more ethical practice. Incorporating training workshops on medical ethics into in-service training programmes conducted by academic colleges, regional level training programmes organized through regional clinical societies most of which have monthly educational sessions and annual conferences could be used as the entry points to provide further training in ethics.

Most respondents (79.2%) agreed that junior doctors tend to follow their consultants’ attitude towards patient care. This reflection denotes the potential for role models such as consultants and other senior doctors to influence their junior doctors to inculcate ethical medical practice. Whilst role models may not necessarily be the most appropriate way to learn medical ethics, medical students and junior doctors do continue to learn many professional attributes from senior doctors and consultants [21], suggesting the need for better awareness of the impact of the hidden curriculum.

Effective doctor-patient communication is a key requirement for ethical medical practice. However, 22.4% of doctors disagreed with the guideline-based statement ‘under no circumstances does a doctor have the right to shout at a patient,’ seemingly indicating an acceptance that there may be instances where a doctor could raise his/her voice when speaking to patients. If some doctors believe so, is it in the best interests of the patients? Do doctors behave authoritatively and expect patients to be submissive? These questions should be explored in a culturally sensitive manner. There have been incidents where patients have specifically stated that hospital staff including doctors are rude and that they do not respect patients resulting even in assaults on hospital staff by patients [22].

Sri Lankan doctors sometimes face an ethical dilemma when managing patients presenting with a history of abortion. In Sri Lanka, abortion is illegal unless the mother’s life is in danger [23]. In this study, 47.3% doctors agreed that the abortions should be legalized, 33.2% disagreed and 19.5% had no opinion. These results indicate that the more inconclusive nature of personal opinions may affect how patients experiencing abortions are managed. On the other hand, Sri Lanka being a multi-ethnic and multicultural society, opinions on legalizing abortions are likely to be influenced by religious opinions.

### Practice of medical ethics

In general, doctors reported that they deliver services ethically ‘always’ and ‘often’. The self-reported practices are more likely to be positively biased due to over reporting ethical practice and under reporting unethical practice. However, study results revealed certain unethical medical practices among doctors.

Examining patients, especially physical examination without the presence of a chaperone has led to a number of allegations against doctors for professional misconduct. In this study only 24.6% doctors reported get a chaperone “sometimes” whereas 3.5% of doctors never obtained the presence of a chaperone. Though the scarcity of staff is a common justification, in most instances, it could be mere negligence especially when doing intimate examinations such as breast, genitalia and rectal examinations. The presence of a chaperone could prevent potential allegations of professional misconduct by doctors and potential harm to patients. The Ayling report on key points of having chaperones stated that across the NHS, there was lack of understanding on the purpose and the use of chaperones. The report recommended that “Trained chaperones should be available to all patients having intimate examinations. Untrained administrative staff or family or friends of the patient should not be expected to act as chaperones” [24].

When prescribing drugs, 40.6% doctors wrote the generic name of the drug alongside the brand name ‘sometimes’; 6.7% never wrote the generic name and only the brand name of the drug. Brand-name prescription contributes to greater out of pocket expenditure; this is already relatively high (50.12% in 2016) in Sri Lanka [25]. High out of pocket expenditure may push households into poverty where the poor (below and near the poverty line) are the most vulnerable [26, 27]. It has been noted that a significant proportion of Sri Lankan doctors including specialists contribute to increasing the costs of medicines [27] leading to further widening of health inequalities. High rates of generic drug use through policy development have resulted in savings of money in developed countries such as United Kingdom, Germany, Netherlands, Canada, United States etc. [28]. However, it was evident from a recent systematic review that sufficient evidence on effective interventions to promote generic prescriptions in low- and middle-income countries were lacking [29].

In this study, 54% doctors responded that they never accept gifts from pharmaceutical companies in recognition of their prescribing pattern while 37.7% said they accepted gifts ‘sometimes’. Accepting gifts given in recognition of prescribing pattern is always an unethical practice based on SLMC medical ethics guidelines. Gifts offered by pharmaceutical companies range from a simple pen to free lunches to international travel expenditure including lodging. The globally recognised significant positive association between drug company sponsored Continuous Medical Education (CME) activities and increased prescribing of sponsors’ medicines [30, 31] may well apply in the Sri Lankan context. Understanding this conflict of interest is very important for eliminating the biased prescription patterns. Evidence suggests that policies against gifts should not be based on the size and the value of the gifts, instead gifts should be prohibited [32]. Even though this might look idealistic, it would be a straightforward policy decision.

Punctuality is a fundamental discipline in any profession, reflecting the characteristics of taking responsibility and truthfulness. Nearly one-fourth of the study population (24.6%) knew two habitual late comers out of five fellow practitioners. Lack of punctuality among doctors has been identified by patients in other south Asian countries as well [33]. The lack of punctuality may be related to problems in work-life balance, burnout and in priority setting, indicating a need for including time management training in CME activities.

### Factors associated with the level of knowledge

There was a significant difference in the mean knowledge score (GHK-51.5, THP-49.1, SBSCH-45.3) between the three institutions studied (*p* = 0.008, ANOVA test). This difference may be due to the lack of CME activities in the newly established teaching hospital included in the study whereas regular CME activities are a feature of the other two hospitals. All three are tertiary care level teaching hospitals where there are many opportunities for doctors to acquire new knowledge. The proportion of postgraduate trainees who had a good knowledge of ethics (60.7%) was higher than the doctors who had no postgraduate training (*p* = 0.02). This is a positive sign that indicates the possibility of improving the knowledge on medical ethics through providing more learning opportunities. There were no significant associations between overall level of knowledge and the factors such as age, sex, religion, duration of the service, marital status and the private practice.

### Limitations

The study instrument was assessed only to establish judgmental validity whereas reliability of the study instrument was not assessed statistically. The results of the study cannot be generalized to all doctors since study participants were selected from only three hospitals based on a convenience sampling method. The potential for selection and information bias regarding under reporting of unethical practice cannot be excluded. Data analysis was carried out using univariate methods, therefore confounding cannot be eliminated.

## Conclusions

Most doctors who participated (81.2%) from these three hospitals had a poor level of knowledge of medical ethics. Postgraduate trainees (60.7%) had a comparatively higher level of knowledge than other doctors (44.4%). The majority (95%) showed a favourable attitude towards gaining knowledge and the need for training. 69.3% were aware of unethical practice among doctors. 12–41% of doctors acknowledged that they ‘sometimes’ engaged in unethical practices related to prescribing drugs, accepting gifts from pharmaceutical companies and obtaining leave. Regular in-service training on medical ethics for doctors could help to improve their knowledge on medical ethics, as well as attitudes and ethical conduct.

## Supplementary information

**Additional file 1.**

## Data Availability

The datasets used and/or analysed during the current study are available from the corresponding author for reasonable requests.

## Abbreviations

SLMC: Sri Lanka Medical Council
GHK: General Hospital Kandy
THP: Teaching Hospital Peradeniya
SBSCH: Sirimavo Bandaranaike Specialised Children’s Hospital Peradeniya
OPD: Out Patient Department
CME: Continuous Medical Education
