# Supplementary material for: Medical ethics: knowledge, attitude and practice among doctors in three teaching hospitals in Sri Lanka
Source: BMC Med Ethics. 2020 Aug 5;21:69. doi: 10.1186/s12910-020-00511-4 (PMC7405426; doi:10.1186/s12910-020-00511-4)
Supplement: Supplementary file 1 — Additional file 1. [file 12910_2020_511_MOESM1_ESM.doc]

# (Confidential)

**Annexure I**

**Study on knowledge, attitudes and practice of medical ethics among medical officers in three teaching hospitals in the Kandy district.**

**August - 2009.**

This survey is carried out to collect information on knowledge, attitudes and practice of medical ethics among medical officers in the aim of providing information about the present situation and improve the knowledge, attitudes and practice of medical ethics.

You are kindly requested to provide responses to the questions asked bellow. **Anonymity will ensure confidentiality of the information provided.** Information will be kept strictly confidential. Please do not write your name on this questionnaire.

**Section - A**

**Please mark " " in the appropriate cage.**

**For Office Use Only**

1. What is your hospital?

1

- - 1. Teaching Hospital Kandy

2

- - 1. Teaching Hospital Peradeniya

3

- - 1. Sirimavo Bandaranaike Specialized Children’s Hospital Peradeniya

2. What is your field of work?

1

1. Administration

**Medical**

2

1. General Medical wards

3

1. Cardiology

4

1. Nephrology

5

1. Neurology

6

1. Dermatology

7

1. Rheumatology

8

1. Chest ward

9

1. Gastroenterology

10

1. Oncology

11

1. Medical ICU

**Surgical**

12

1. General Surgical

13

1. ENT

14

1. Eye

15

1. Oncosurgery

16

1. Neurosurgery

17

1. Surgical ICU
2. Paediatric Surgery

1ccept private treatment8

1. Plastic Surgery

19

1. Cardio Thoracic

20

**Paediatric**

21

o youimes conductsical

1. General Paediatric

22

1. Neonatology

23

1. SBU/PB

24

1. Pediatric ICU

**Other Units**

1. Obstetrics and Gynecology

25

1. Anesthesiology

26

1. Pathology/Haematology

27

1. Radiology

28

1. Psychiatry

29

30

1. OPD

31

1. ETU

32

1. PCU

33

1. Medico legal

34

1. **Any Other Unit (please specify)**
2. What is your current professional status?

1

1. Special grade medical officer (consultant)

2

1. Medical officer - Grade 1

3

1. Medical officer - Grade 2

4

1. Preliminary grade medical officer
2. What is your date of birth?

|  |  |  |  |  |  |  |  |
| --- | --- | --- | --- | --- | --- | --- | --- |
| D | D | M | M | Y | Y | Y | Y |

1. What is your sex?

1

1. Male

2

1. Female
2. To what ethnic group do you belong?

1

1. Sinhalese

2

1. Tamil

3

1. Moor

4

1. Other (please specify)
2. What is your religion?

1

1. Buddhist

2

1. Hindu
2. Roman Catholic

3

1. Christian

5

1. Protestant

6

4

1. Islam

7

1. Other (please specify)

**For Office Use Only**

1. What is your current marital status?

1

1. Married

2

1. Separated

3

1. Divorced

4

1. Widowed

5

1. Single
2. Are you a postgraduate trainee?

1

- 1. Yes

2

- 1. No

1. For how long have you been working in the health services?

| 1. 0 – 4 years |  | 1 |
| --- | --- | --- |
| 1. 5 – 9 years |  | 2 |
| 1. 10 – 14 years |  | 3 |
| 1. 15–20 years |  | 4 |
| 1. > 20 years |  | 5 |

1. Are you engaged in private medical practice?

1

1. Yes

2

1. No

**Section - B**

**Please state whether the following statements are true or false. Mark with a “ ” in the relevant cage.**

1. There is a written document which provides guidelines on ethical conduct for Sri Lankan doctors.

1

1. True

2

1. False

3

1. Don’t Know
2. The Nuremberg Code and the Declaration of Helsinki are important landmarks in the evolution of research ethics.

1

1. True

2

1. False

3

1. Don’t Know
2. A child aged 12-18 years can give informed consent for treatment or a medical procedure provided he or she is competent to understand the nature, purpose and the possible consequences of the intervention.

1

1. True

2

1. False

3

1. Don’t Know
2. The doctor should take informed consent from the parent of a child, aged 12-18 years, for treatment or procedure.

1

1. True

2

1. False

3

1. Don’t Know
2. If the parents have never been married, only the mother has parental responsibility to consent for treatment of the child.

1

1. True

2

1. False

3

1. Don’t Know
2. When writing prescriptions full signature and the address of the physician is a must.

1

1. True

2

1. False

3

1. Don’t Know
2. When prescribing a drug in its brand name it should always be in parenthesis after the generic name.

1

1. True

2

1. False

3

1. Don’t Know
2. No doctors should advertise his/her successes in treatment.
3. True

2

1

1. False

3

1. Don’t Know
2. Those who write articles or are interviewed by the press, should refrain from publishing their photographs

1

1. True

2

1. False

3

1. Don’t Know
2. In television and radio broadcasts, the announcing of names, specialties, registered qualifications of participants should be confined only to the commencement and the end of the programme.

1

1. True

2

1. False

3

1. Don’t Know
2. Any payment to a healthy research participant should be for expenses, time, inconveniences or discomfort, and never for the risk.

1

1. True

2

1. False

3

1. Don’t Know
2. It is considered unethical for a doctor to own a pharmacy for dispensing prescriptions by doctors other than him/her self or for sale of medical or surgical appliances.

1

1. True

2

1. False

3

1. Don’t Know
2. Individual gifts of minimal value from pharmaceutical companies are permissible as long as they serve an immediate purpose, e.g. writing pads and pens.

1

1. True

2

1. False

3

1. Don’t Know
2. Physicians should not accept any gifts from pharmaceutical companies if they are given in recognition of their prescribing practices.

1

1. True

2

1. False

3

1. Don’t Know
2. Contributions from pharmaceutical companies to medical organizations, to defray costs of continuous medical education, conferences, professional meetings, workshops, seminars etc. are permissible.

1

1. True

2

1. False

3

1. Don’t Know
2. It is a responsibility of a doctor to bring instances of professional misconduct, incapability, dishonesty, or negligence of a fellow medical practitioner to the notice of the Sri Lanka Medical Council in the best interests of the medical profession and the general public.

1

1. True

2

1. False

3

1. Don’t Know
2. The doctor is perfectly entitled, perhaps as a duty, to disclose information if asked to do so by the patient or his legal adviser, with a valid consent for disclosure.

1

1. True

2

1. False

3

1. Don’t Know
2. A doctor’s signature is required by statute (law) on certificates for a variety of purposes, on the presumption that the truth of any statement which a doctor may certify can be accepted without question.

1

1. True

2

1. False

3

1. Don’t Know
2. Neither AIDS nor HIV status are statutorily (legally) notifiable conditions.

1

1. True

2

1. False

3

1. Don’t Know

1. If a patient infected with HIV refuses to give consent or declines to disclose information to the spouse or the sexual partner, the doctor may disclose information after informing the patient, only if he is the physician for both.

1

1. True

2

1. False

3

1. Don’t Know
2. It is the clinician in charge of the care of patients, is ethically responsible for the confidentiality of medical records.

1

1. True

2

1. False

3

1. Don’t Know
2. If the doctor examines a patient at the request of the police, informed written consent from the patient is not necessary to pass on information to the police.

1

1. True

2

1. False

3

1. Don’t Know
2. Doctor’s conviction of a criminal offence which is not directly connected with the profession can lead to disciplinary proceedings at the medical council.

1

1. True

2

1. False

3

1. Don’t Know
2. Where the law allows therapeutic abortion to save the life of the mother, if there is any religious or moral objection to abortion by a doctor, he/she cannot be forced to perform or assist in the termination of pregnancy.

1

1. True

2

1. False

3

1. Don’t Know
2. If the criminal abortion was performed by the women herself, it is unethical for a doctor to report this patient to the police or to anyone else unless the patient’s life is in danger or death occurs.

2

3

1

1. True
2. False
3. Don’t Know

**Section - C**

**Different medical officers have different opinions on medical ethics. Please indicate if you agree or disagree with the following statements.**

**Rate your reaction to each statement by marking “ ” in the appropriate cage.**

1. I like my job more than any other job.

2

3

1

4

5

1. Agree strongly
2. Agree
3. No opinion
4. Disagree
5. Disagree strongly
6. At present the extent of ethical medical practice among doctors is satisfactory.

2

3

1

4

5

1. Agree strongly
2. Agree
3. No opinion
4. Disagree
5. Disagree strongly
6. In my opinion, the extent of teaching on medical ethics in the undergraduate curriculum is not adequate.

2

3

1

4

5

1. Agree strongly
2. Agree
3. No opinion
4. Disagree
5. Disagree strongly
6. Doctors should make certain that their actions do not intentionally harm another even to a small degree.

2

3

1

4

5

1. Agree strongly
2. Agree
3. No opinion
4. Disagree
5. Disagree strongly
6. Doctors should never harm another person physically or psychologically

2

3

1

4

5

1. Agree strongly
2. Agree
3. No opinion
4. Disagree
5. Disagree strongly
6. Doctors should not perform an action which might in anyway threaten the dignity of another individual.

2

3

1

4

5

1. Agree strongly
2. Agree
3. No opinion
4. Disagree
5. Disagree strongly
6. Doctors should treat patients as they would wish others to treat them if they were the patients.

2

3

1

4

5

1. Agree strongly
2. Agree
3. No opinion
4. Disagree
5. Disagree strongly
6. Under no circumstances, a doctor has right to shout at a patient.

2

3

1

4

5

1. Agree strongly
2. Agree
3. No opinion
4. Disagree
5. Disagree strongly
6. The quality of the service of doctor in the government hospital is negatively affected by his/her private practice.

2

3

1

4

5

1. Agree strongly
2. Agree
3. No opinion
4. Disagree
5. Disagree strongly
6. Juniors tend to follow their consultant’s attitudes towards patient care.

2

3

1

4

5

1. Agree strongly
2. Agree
3. No opinion
4. Disagree
5. Disagree strongly
6. Favouritism for students in medical exams is rare.

2

3

1

4

5

1. Agree strongly
2. Agree
3. No opinion
4. Disagree
5. Disagree strongly
6. Having an emotional or sexual relationship with a patient (or with a member of the patient’s family), even with consent, is unethical.

2

3

1

4

1. Agree strongly
2. Agree
3. No opinion
4. Disagree

5

1. Disagree strongly
2. Abortion should be legalized in Sri Lanka.

2

3

1

4

1. Agree strongly
2. Agree
3. No opinion
4. Disagree

5

1. Disagree strongly
2. Strikes done by doctors are indirectly beneficial to patients.

2

3

1

4

1. Agree strongly
2. Agree
3. No opinion
4. Disagree

5

1. Disagree strongly
2. In-service training on medical ethics is a necessity for doctors.

2

3

1

4

1. Agree strongly
2. Agree
3. No opinion
4. Disagree

5

1. Disagree strongly

Section – D

**Please indicate your responses according to your medical practice.**

**Rate your reaction to each statement by marking “ ” in the appropriate cage.**

1. I treat every patient considerately.

2

3

1

4

1. Always
2. Often
3. Some times
4. Never
5. When examining a patient I get a chaperone.

2

3

1

4

1. Always
2. Often
3. Some times
4. Never
5. I spend enough time to explain the nature, purpose and possible consequences of treatment or procedure when obtaining informed consent from patients.

2

3

1

4

1. Always
2. Often
3. Some times
4. Never
5. When prescribing drugs in their brand names, I write the generic name also.

2

3

1

4

1. Always
2. Often
3. Some times
4. Never
5. On average, when you consider five (5) of your fellow practitioners who are currently working with you, how many of them are habitually late for the duty? Please mark “ ” in the cage provided.

2

3

1

4

5

1. Only one
2. Two
3. Three
4. Four
5. All five

6

1. None

1. As a medical officer I dress appropriately.

2

3

1

4

1. Always
2. Often
3. Some times
4. Never
5. I engage in continuous medical education (CME) activities.

2

3

1

4

1. Always
2. Often
3. Some times
4. Never
5. When taking leave, I follow the exact rules and regulations.

2

3

1

4

1. Always
2. Often
3. Some times
4. Never
5. I engage in private medical practice during normal working hours.

2

1

1. Always
2. Often

3

1. Some times

4

1. Never
2. Do you influence patients directly or indirectly to accept private treatment.

2

1

1. Always
2. Often

3

1. Some times

4

1. Never
2. When you come across an instance of professional misconduct, do you bring it to the notice of the medical council?

2

1

1. Always
2. Often

4

3

1. Some times
2. Never
3. Do you accept gifts from pharmaceutical companies, given in recognition of your prescribing pattern?

2

1

1. Always
2. Often

4

3

1. Some times
2. Never
3. When managing patients, I consider patient’s religious and cultural views.

2

1

1. Always
2. Often

4

3

1. Some times
2. Never
3. On average how would you describe your alcohol consumption pattern?

1

1. Consumed daily

2

1. Consumed once a week

3

1. Consumed once a month

4

1. Occasionally

5

1. Never consumed
2. On average how would you describe your smoking pattern?

1

1. Daily

2

1. Once a week

3

1. Once a month

4

1. occasionally

5

1. Never smoked

**Thank you for spending your valuable time to fill this questionnaire.**
